# A Multicenter, Randomized, Controlled Trial of Electroacupuncture for Perimenopause Women with Mild-Moderate Depression

**DOI:** 10.1155/2018/5351210

**Published:** 2018-05-29

**Authors:** Sheng Li, Zhao-Feng Li, Qian Wu, Xiao-Chuan Guo, Zhen-Hua Xu, Xiao-Bin Li, Rong Chen, Dao-you Zhou, Cong Wang, Quan Duan, Jian Sun, Ding Luo, Min-Ying Li, Jun-Ling Wang, Hui Xie, Li-Hua Xuan, Sheng-Yong Su, Dong-Mian Huang, Zhi-Shun Liu, Wen-Bin Fu

**Affiliations:** ^1^Department of Acupuncture and Moxibustion, The Second Affiliated Hospital of Guangzhou University of Chinese Medicine, Guangzhou, Guangdong, China; ^2^Postdoctoral Mobile Station, Guangzhou University of Chinese Medicine, Guangzhou, Guangdong, China; ^3^Department of Psychology, Sun Yat-Sen University, Guangzhou, China; ^4^Department of Gynecology, Guangzhou University of Chinese Medicine, Guangzhou, Guangdong, China; ^5^Department of Neurology, Guangzhou University of Chinese Medicine, Guangzhou, Guangdong, China; ^6^Department of Traditional Therapy, Guangzhou University of Chinese Medicine, Guangzhou, Guangdong, China; ^7^Department of Traditional Chinese Medicine, Shenzhen Maternity & Child Healthcare Hospital, Shenzhen, Guangdong, China; ^8^Department of Rehabilitation, Chenzhou No. 1 People's Hospital, Chenzhou, Hunan, China; ^9^Department of Acupuncture, The First Affiliated Hospital of Zhejiang Chinese Medical University, Hangzhou, Zhejiang, China; ^10^Department of Acupuncture and Moxibustion, The First Affiliated Hospital of Guangxi University of Chinese Medicine, Nanning, Guangxi, China; ^11^Department of Acupuncture and Rehabilitation, Traditional Chinese Medicine Hospital of Hainan Province, China; ^12^Department of Acupuncture and Moxibustion, Guang'an Men Hospital, China Academy of Chinese Medical Sciences, Beijing, China

## Abstract

**Objective:**

Up to 62% of perimenopausal women have depression symptoms. However, there is no efficacy treatment. The aim of this study is to compare the clinical efficacy and safety of EA therapy and escitalopram on perimenopause women with mild-moderate depressive symptom.

**Method:**

A multicenter, randomized, positive-controlled clinical trial was conducted at 6 hospitals in China. 242 perimenopause women with mild-moderate depressive symptom were recruited and randomly assigned to receive 36 sessions of EA treatment or escitalopram treatment. The primary outcome measure was the 17-item Hamilton Depression Rating Scale (HAMD-17). The secondary outcome measures include menopause-specific quality of life (MENQOL) and serum sexual hormones which include estrogen, follicle-stimulating hormone, and luteinizing hormone.

**Results:**

221 (91.3%) completed the study, including 116 in the EA group and 105 in the escitalopram group. The baseline levels of demographic and outcome measurements were similar in the two groups. In the intervention period, there was no difference between two groups. However, in the follow-up, both HAMD-17 and MENQOL were significantly decreased, and at week 24 the mean differences were −2.23 and −8.97, respectively. There were no significant differences in the change of serum sexual hormones between the two groups. No serious adverse events occurred.

**Conclusion:**

EA treatment is effective and safe in relieving depression symptom and improving the quality of life in the perimenopausal depression. Further research is needed to understand long-term efficacy and explore the mechanism of this intervention. This study is registered with* ClinicalTrials.gov *NCT02423694.

## 1. Introduction 

Depression is the most common psychiatric disorder and the leading cause of disease-related disability in women, especially in perimenopause [1, 2]. Perimenopause is the transitional phase to nonreproductive life. During this period the reproductive hormone fluctuates because of ovarian follicular function. However, the changes of hormone are associated with depressive symptom [3–5]. In China, the depression, with high incidence rate of 61.88%, is one of the most frequent symptoms during menopausal transition [6], which made significant effects on individual's life.

Hormone therapy (HT) has been as mainstream treatment for perimenopause symptoms for many years [7]. HT is commonly used to alleviate climacteric symptoms, but its effects to depression symptom are still not questionable. Recently a narrative review showed that the progestogen component in combined hormone therapy was less effective in perimenopause depression women and even induce negative mood symptoms [8]. In case of more severe condition, combination of hormone therapy and antidepressants was considered. Growing research has shown that long-term HT increases the risk of cancer [9, 10], heart disease [11], and stroke [12]. In addition, antidepressants such as escitalopram were widely used to improve depression but had drug dependence and frequent side effects, such as dizziness, fatigue, insomnia, constipation, and limited wide use [13]. Consequently, safe and effective therapy was urgently needed for perimenopause with depression.

Although numbers of randomized controlled trials have shown that electroacupuncture (EA) was effective and safe on perimenopause related symptoms such as vasomotor and sleep disturbance [14–21], few studies mentioned that acupuncture could ameliorate depression [14, 16, 17, 21]. As far as we know, there were no detailed investigations about the efficacy of acupuncture on depression symptom in perimenopause. In order to examine the clinical efficacy and safety of EA on mild-to-moderate depression symptom in perimenopause women, a multicenter, randomized, positive-controlled trial was conducted. We hypothesized that EA could be superior in reducing the depression symptom and improving quality of life to antidepressant escitalopram in perimenopause with depression symptoms women.

## 2. Methods

### 2.1. Design Overview

Multicenter, randomized, escitalopram-controlled clinical trial was conducted at 6 hospitals in China. The trial duration per patient was 26 weeks: 2 weeks before randomization; 12 weeks of intervention; and 12 weeks (week 12–week 24) of follow-up without any intervention. The clinical trial results will be reported according to the CONSORT guidelines and the Standards for Reporting Interventions in Clinical Trials of Acupuncture (STRICTA) guidelines [22]. The Institution Ethics Committee of Guangdong Provincial Hospital of Traditional Chinese Medicine provided ethics approval (B2014-008-01).

### 2.2. Participants

Participants were recruited from hospitals and communities via poster and newspaper advertisements in different hospitals.

#### 2.2.1. Inclusion Criteria

Participants were included if they (1) are deemed perimenopause according to the 2012 criteria of the North American menopause society [23] (Supplement Figure 1(s)); (2) they meet the criteria of DSM-5™ [24] and ICD-10 [25] for the mild-moderate depression; (3) the total score of the 17-item Hamilton Depression Rating Scale (HAMD-17) is <23 and ≥8; (4) they were between the ages of 45 and 55; (5) they had not undergone any hormone therapy or antidepressant during the past 3 months before enrollment; (6) they are voluntarily participating in this trial with a written informed consent form.

#### 2.2.2. Exclusion Criteria

Participants with the following conditions will be excluded: (1) suicidal ideation assessed by the Beck depression inventory; (2) SCL-90 score > 26; (3) use of estrogen, selective serotonin reuptake inhibitors, soybean isoflavone, vitamin E, or black sesame in the past 4 weeks; (4) being allergic to escitalopram; (5) smoking or heavy alcohol intake; (6) presence of a cardiac pacemaker or artificial joint; (7) desire to become pregnant or pregnant or breastfeeding women; (8) mandatory indication for HT.

### 2.3. Randomization

Participants were allocated to the EA group or escitalopram group using a central randomization system for clinical trial using a 1 : 1 ratio. The random sequences were generated by using SAS software. The outcome assessors and statisticians were blinded to treatment allocation.

### 2.4. Interventions

The study interventions were according to the classical principles of traditional Chinese medicine and the details are described in accordance with the STRICTA 2010 extension [22]. All acupuncturists had at least 5 years of acupuncture experience. Hwato brand disposable needles (0.3 *∗* 0.4 mm, 0.3 *∗* 0.25 mm, and 0.3 *∗* 0.5 mm) and SDZ-V electroacupuncture apparatuses were used.

Participants in the EA group received acupuncture at Guanyuan (RN4), Zigong (EX-CA1, bilateral), Tianshu (ST25, bilateral), Sanyingjiao (SP6, bilateral), Hegu (LI4, bilateral), Taichong (LR3, bilateral), Baihui (DU20), and Yintang (EX-HN3). After skin disinfection, the needles were vertically inserted into the above points except DU20 and EX-HN3, by tube-guide method. In DU20 and EX-HN3 point, needle was obliquely inserted with 30° angle. The point was stimulated manually until patients feel heaviness, soreness, distension, or numbness sensation (deqi). This is important for acupuncture efficacy. Paired electrodes from the EA apparatus were attached to the needle handles at bilateral EX-CA1, ST 25, and DU20, EX-HN3. The EA stimulation was retained 30 min with a dilatational wave of 50 HZ and a current intensity of 0.5~1 mA. Participants received 3 treatment sessions per week for 12 consecutive weeks, 36 sessions in total.

Participants in escitalopram group received a once daily 10 mg escitalopram for 12 weeks.

Through the trial, the participants were treated separately to avoid communication and were discouraged from receiving any other antidepression treatment, and if they have to receive other treatments, they will be asked to document them.

### 2.5. Outcomes and Follow-Up

The primary outcome was the change of depression severity score, which was measured by the HAMD-17. This questionnaire was completed by each participant at baseline and weeks 4, 8, 12, 16, and 24. The secondary outcome includes the change of health related quality of life and sexual hormones. Health related quality of life was measured by the menopause-specific quality of life questionnaire (MENQOL) and completed by each participant at baseline and weeks 4, 8, 12, 16, 20, and 24. Sexual hormones including FSH, LH, and E2 were measured by a fully automatic chemiluminescence instrument. Note that the blood samples in menopausal transition were obtained at the last menstrual period days 2–5 at baseline and week 12. Adverse events were assessed by the acupuncturists or investigator, and severe adverse events were reported to the principal investigator within 24 hours after occurrence.

### 2.6. Statistical Analysis

Outcome data was analyzed according to the intention-to-treat principle, with all randomly assigned participants after baseline assessment regardless of received intervention or not. Missing data were replaced with the data from the latest assessment. The outcome data were analyzed with SPSS (version 22.o; SPSS Inc., Chicago, IL, USA). Descriptive statistics (*T*-test, nonparametric test, and Chi square test) were used to summarize demographic features between the two groups. The changes from baseline in HAMD-17 and MENQOL were analyzed by repeated-measures analysis of variance (ANOVA), using group and site as fixed factor. If the main interaction effect was evident for the outcomes, least significant difference test was performed to compare within/between two groups for the same time points, baseline and posttreatment. Nonparametric test was used to analyze the sexual hormones levels. Tests were two-sided, and *P* < 0.05 was considered statistically significant.

### 2.7. Results

**Figure 1:**
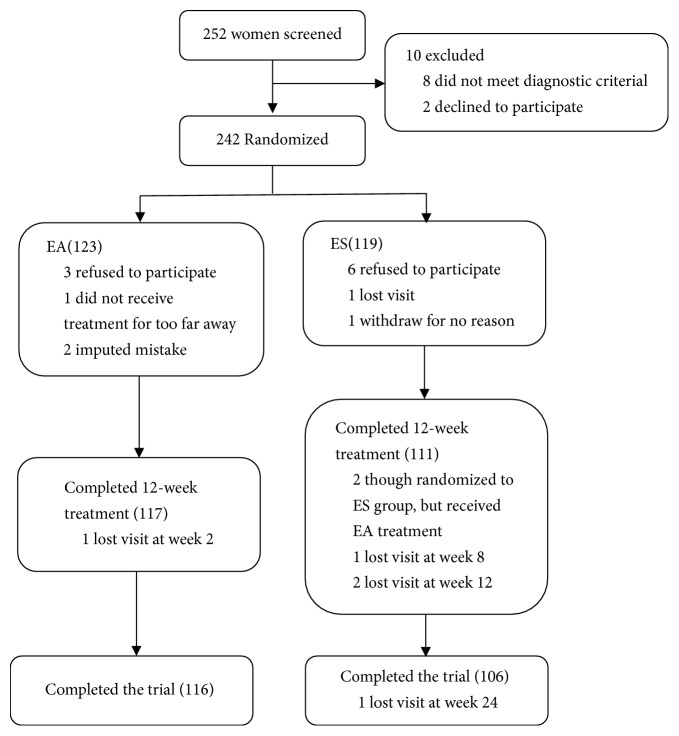
Study flow diagram. EA means electroacupuncture treatment; ES means escitalopram treatment.

Between October 10, 2013, and October 31, 2015, we screened 252 participants for eligibility, of whom 242 were randomly assigned to receive EA (*n* = 123) or escitalopram (*n* = 119) treatment. Of these, 221 (91.3%) completed the treatment and follow-up visit. A total of 20 (8.3%) participants dropped out during the study: 7 (2.9%) in the EA group and 13 (5.4%) in the ES group. Disposition and reasons for exclusion or discontinuation were shown in Figure 1. The baseline characteristics were similar between two groups (Table 1).

#### 2.7.1. HAMD-17

**Figure 2:**
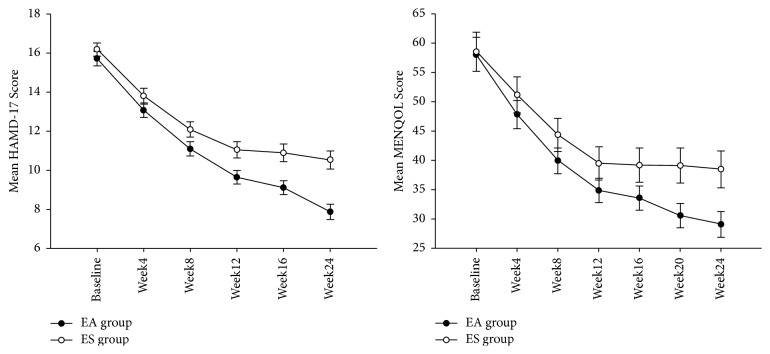
HAMD-17 score and MENQOL score at baseline and weeks 4, 8, 12, 16, 20, and 24. Error bars represent standard error. EA means electroacupuncture treatment; ES means escitalopram treatment.

As shown in Table 1 and Figure 2, the baseline HAMD-17 scores were similar in the two groups. After 12-week treatment, the HAMD-17 score in the two groups decreased, but there was no difference between the two groups, until week 16. At week 24, the change from baseline in HAMD-17 was −7.84 (SD, 4.83) in the EA group and −5.65 (SD, 4.26) in the ES group (between-group difference, −2.23 [CI, −3.31 to −1.15]; *P* < 0.001) (Table 2). Though the multivariate test showed that there was significant main effect in center (*P* < 0.001), the interaction effect between center and group was nonsignificant (*P* = 0.687), suggesting that the treatment effect of the centers was homogeneous.

#### 2.7.2. MENQOL 

As shown in Table 1 and Figure 2, the MENQOL scores were similar at baseline. After 12-week treatment, the HAMD-17 score in the two groups decreased, but there was no difference between the two groups, until week 16. At week 24, the change from baseline in MENQOL was −28.94 (SD, 23.22) in the EA group and −20.04 (SD, 18.98) in the ES group (between-group difference, −8.97 [CI, −14.12 to −3.81]; *P* = 0.001). The multivariate test showed that there was significant main effect in center (*P* < 0.001), but the interaction effect between center and group was nonsignificant (*P* = 0.402), suggesting the treatment effect of the centers was homogeneous.

#### 2.7.3. Sexual Hormones

Sexual hormones as shown in Tables 1 and 3, FSH, LH, and E_2_ were similar in the two groups at baseline. No differences were found in FSH, LH, and E2 levels between groups or in groups after treatment.

### 2.8. Safety

All enrolled subjects who received either EA or escitalopram treatment were included in the safety population. EA was generally well tolerated. 14 participants in EA group and 18 (one participant could report multiple adverse event) in escitalopram reported adverse effects during the intervention period, and no serious adverse events reported. The adverse events reported in EA group was subcutaneous hematoma. While the adverse events in escitalopram were various, the most common events were dizzy, palpitation, and stomachache.

## 3. Discussion

The findings from our multicenter, randomized, positive-controlled trial showed that a 12-week course of EA treatment significantly relieved mild-moderate depressive symptom and improved the quality of life in perimenopause. And the long-term effect of EA was superior to escitalopram. The sexual hormones showed no significantly within group or between group, before or after treatment. The incidence of adverse events was tolerable.

Escitalopram is an antidepressant of the selective serotonin reuptake inhibitor (SSRI), which is widely used in perimenopause with depression. The escitalopram like other SSRIs has side effects such as headache, nausea, insomnia, and somnolence, which had low patient's compliance. Our study showed that after 12-week intervention, both EA and escitalopram significantly reduced the HAMD-17 score. But in the follow-up, the changes of HAMD-17 score in EA group were more significant than escitalopram, suggesting that EA has nice long-term antidepression effect. As I know this is the first study using positive control (escitalopram) to evaluate the EA antidepression. The previous studies most were sham-EA or placebo-control design [15, 19]. Our study showed that EA treatment has equal antidepression effect with escitalopram, in the intervention period. What is more, the long-term effect was more superior in EA group, suggesting that EA could be used as potential therapy for perimenopause with depressive symptom.

The MENQOL, a 29-item questionnaire covering 4 areas of vasomotor, psychosocial, physical, and sexual functioning, is a well-accepted international questionnaire in assessing menopause symptoms. In this study, after 12-week intervention, the MENQOL score in EA and escitalopram group were both declined compared with baseline but showed no difference between the two groups. In the follow-up, the change of MENQOL in EA group was more significant than escitalopram group, suggesting that EA has better effect in long term to improve the quality of life. These was similar to previous studies that EA could improve the menopause quality of life [19, 26]. Compared to the previous study, higher scores of MENQOL in baseline were found in this trial, even though EA effectively improved life quality.

Depression symptom is closely associated with menopausal stage and sexual hormone changes [4, 27]. Zhou et al. [28] found that acupuncture improves the hot flashes in bilaterally ovariectomized women which has relationship with modulated FSH and E2 level. In addition, there are 2 metareviews showing that acupuncture is beneficial for polycystic ovarian syndrome that has association with modulated LH, LH/FSH, and testosterone [29, 30]. Despite this, whether the acupuncture beneficial for perimenopause with depression is associated with sexual hormones remained unclear. Thus, in this study, we measured the sexual hormones, but there were no differences found within group or between group, before and after treatment. This was consistent with previous studies [31]. Several reasons may explain it. First, during the perimenopause, the decline of E2 and increase of FSH and LH are an irreversible physiological process. EA could not obviously resist this natural tendency. Second, the perimenopause depressive symptom is complex and not entirely hormone based. EA therapy may relieve depression through other mechanisms, such as upregulating 5-HT [32], EAAT2 [33], and PKA/CREB [34] expression. In addition, the mental support could also influence depressive symptom. Thus, further research is needed to confirm the mechanism of EA treatment to improve depressive perimenopause symptom. Note that the standard deviation in the hormones was large. The following reasons may responsible for it. First, the perimenopause includes early menopausal transition, late menopausal transition, and early postmenopause [23] (Supplementary Figure 1); the hormone was greatly different in those 3 stages. We also analyzed the hormones changes by perimenopause stage, and they still show no difference. Second, it was multicenter trial, which may increase error.

There were several limitations in this study. First, a placebo-control (sham-EA) group was lacking in this study, which made it hard to distinguish whether it was placebo effect or not. Some studies reported that acupuncture was more effective than no treatment. However, compared with sham group it showed that it was not efficacious [21, 35]. Second, there was no follow-up beyond 24 weeks; a long-term follow-up was needed. Third, although we tried our best to control and reduce bias, potential bias was still unavoidable, since it was unblended clinical trial. Participants in the EA group were vulnerable to interact their therapists and receive mental support compared to those in the escitalopram group. Finally, the therapeutic evaluation of EA therapy for perimenopause with depression symptom was mainly based on questionnaires. More objective biochemical markers are still insufficient.

In conclusion, 12 weeks of EA treatment decreased the depressive symptom and improved quality of life in perimenopause but did not change the sexual hormones. The biological mechanisms under the therapeutic effects of EA in perimenopause remain to be further explored.

## Figures and Tables

**Table 1:** Baseline characteristics of participants.

Demographic variables	EA (*n* = 116)	Control (*n* = 105)
Age, mean (SD), y	49.83 (3.1)	49.93 (3.1)
BMI, mean (SD)	21.27 (4.0)	21.09 (4.0)
Race (%)		
*Han*	110 (94.0)	107 (96.4)
*Minorities*	7 (6.0)	4 (3.6)
Marital status (%)		
*Married*	116 (99.1)	109 (98.2)
*Unmarried*	1 (0.1)	0 (0)
*Widowed*	0 (0)	2 (0.8)
Occupational status (%)		
*Employed*	55 (47.4)	51 (48.6)
*Unemployed*	45 (38.8)	40 (38.1)
*Pensioner*	16 (13.8)	14 (13.3)
Educational level (%)		
*Primary education or less*	3 (2.6)	12 (10.8)
*Secondary education*	60 (51.3)	61 (54.9)
*Tertiary education*	54 (46.2)	38 (34.2)
Course, mean (SD), m	20.6 (16.2)	20.2 (16.5)
Perimenopause stage (%)		
*Early menopausal transition*	56 (47.9)	45 (40.5)
*Late menopausal transition*	24 (20.5)	28 (25.2)
*Early postmenopause*	37 (31.6)	38 (34.2)
HAMD, mean (SD)	15.72 (4.02)	16.19 (3.57)
MENQOL, mean (SD)	58.07 (31.76)	58.58 (34.84)
Sexual hormones		
*FSH (pmol/L)*	42.45 (36.84)	54.57 (98.53)
*LH (IU/L)*	22.45 (17.58)	27.92 (46.48)
*E2 (IU/L)*	179.41 (244.58)	140.26 (199.25)

BMI = body mass index.

**Table 2:** The change from baseline of HAMD-17 and MENQOL score.

Outcome	EA (*n* = 116)	ES (*n* = 105)	Between-group differences (95% CI)	*P*
Change from baseline in HAMD (Mean ± SD)				
Week 4	−2.64 ± 3.10	−2.38 ± 2.76	−0.25 (−0.99 0.48)	0.498
Week 8	−4.62 ± 3.65	−4.10 ± 3.64	−0.55 (−1.45 0.34)	0.226
Week 12	−6.07 ± 4.68	−5.13 + 4.48	−1.03 (−2.08 0.02)	0.055
Week 16	−6.60 ± 4.58	−5.30 ± 4.38	−1.36 (−2.41 −0.31)	0.012
Week 24	−7.84 ± 4.83	−5.65 ± 4.26	−2.23 (−3.31 −1.15)	0.000
Change from baseline in MENQOL (Mean ± SD)				
Week 4	−10.21 ± 13.73	−7.39 ± 8.62	−2.84 (−5.76 0.08)	0.057
Week 8	−18.09 ± 17.97	−14.16 ± 13.63	−3.95 (−7.89 −0.01)	0.050
Week 12	−23.18 ± 22.45	−19.03 ± 17.25	−4.32 (−9.17 0.52)	0.080
Week 16	−24.44 ± 22.44	−19.34 ± 18.08	−5.20 (−10.14 −0.28)	0.039
Week 20	−27.43 ± 22.64	−19.42 ± 18.65	−8.12 (−13.19 −3.06)	0.002
Week 24	−28.94 ± 23.22	−20.04 ± 18.98	−8.97 (−14.12 −3.81)	0.001

EA means electroacupuncture treatment; ES means escitalopram treatment. Data is given as the mean ± standard deviation.

**Table 3:** The change from baseline of sexual hormone levels.

Sexual hormones	EA (*n* = 94)	ES (*n* = 85)	*Z*	*P*
FSH (pmol/L)	−0.99 ± 25.74	−12.37 ± 102.72	−0.699	0.485
LH (IU/L)	−0.05 ± 14.42	−6.59 ± 48.09	−0.605	0.545
E2 (IU/L)	−33.20 ± 249.22	6.32 ± 294.55	−0.478	0.633

Using Mann–Whitney *U* test.

## Data Availability

All data and materials are described within the article.

## Supplementary Materials

Supplementary Materials Supplementary Figure 1: perimenopause stage.
