# Supplementary figures and images for: A Multicenter, Randomized, Controlled Trial of Electroacupuncture for Perimenopause Women with Mild-Moderate Depression
Source: Biomed Res Int. 2018 May 29;2018:5351210. doi: 10.1155/2018/5351210 (PMC5996410; doi:10.1155/2018/5351210)

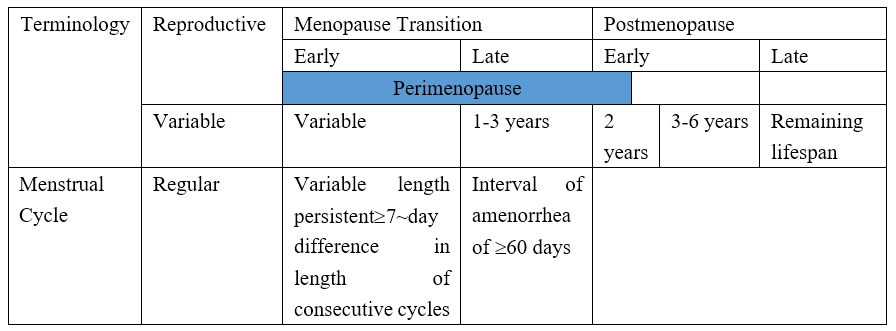

Supplement: Supplementary Materials — Supplementary Figure 1: perimenopause stage. [file 5351210.f1.tif]
